# Experimental Study on Strength Enhancement of Expansive Grout

**DOI:** 10.3390/ma15030885

**Published:** 2022-01-24

**Authors:** Di Wang, Yicheng Ye, Nan Yao, Yiming Liu, Xingmin Deng

**Affiliations:** 1 School of Resources and Environmental Engineering, Wuhan University of Science and Technology, Wuhan 430081, China; 18571545679@163.com (D.W.); yeyicheng@wust.edu.cn (Y.Y.); yuyuzi2734@163.com (Y.L.); zxx970622@163.com (X.D.); 2 Hubei Key Laboratory for Efficient Utilization and Agglomeration of Metallurgic Mineral Resources, Wuhan 430081, China

**Keywords:** expansive grout, quartz sand, expansion behavior, UCS

## Abstract

Adding an expansive agent to ordinary grout can cause an expansion in volume, but also reduces its strength. In order to improve the strength of expansive grout, quartz sand is used as the strength enhancement additive. In this study, the expansion behavior and mechanical properties of the expansive grout with quartz sand are explored, through expansion development monitoring, uniaxial compression strength (UCS), acoustic emission (AE), SEM and XRD test methods. The results showed that: (1) The final expansion ratio and expansion development of the samples are related to the use of an expansive agent, but not affected by quartz sand. With the increase in expansion agent content, the average expansion ratios of the samples are 0.03%, 0.16%, 0.67%, 1.06% and 1.48%; (2) The UCS of the samples decreases with the increase in expansive agent content but increases with the increase in quartz sand content. Compared with no quartz sand, and with the increase in quartz sand content, the average strength of the samples increased by 10.51%, 29.88%, and 37.92%; (3) Quartz sand does not effectively participate in the hydration reaction, but it can effectively enhance the strength of the expansive grout without affecting its volume expansion, which makes it an ideal expansive grout strength enhancement additive.

## 1. Introduction

During the construction of underground roadways, fractured rock masses are often encountered, and the stability of the fractured rock masses becomes an obstacle to the tunnel support engineering [1,2]. Grouting reinforcement technology is often used in engineering. Cement grout or other types of grout are injected into fissures by drilling holes in order to achieve the effect of strengthening and maintaining the stability of the tunnel [3,4]. Grouting can improve the adhesion and friction of the fracture surface and improve the integrity of the roof surrounding rock [5,6,7,8,9,10]. The ordinary grout only provides a "bonding" effect on the rock mass and requires a high degree of cohesive force produced by the grout. At the same time, because the grout is usually an ordinary cement material, its shrinkage may affect the grouting effect [11,12]. 

In response to this problem, an expansive grout using an expansive agent has been developed. Expansive grout is mainly used to solve the problem of roadway grouting support in the steeply inclined strata of a weak bedding plane. Compared with ordinary grout, which only provides a bonding effect, the expansive grout produces restraint stress through volume expansion, which can achieve a superior grouting effect. [13,14]. Due to the action of the expansive agent, the volume of grout will expand during the hydration process [15,16]; the constrained stress generated by the volume expansion of the grout causes the fractured rock masses in the grouting area to squeeze together, and then a complete stone mass is formed under the bonding action of the expansive grout, resulting in a “squeezing before bonding” grouting reinforcement effect, which improves the overall reinforcement strength [14,17,18].

From the performance of grout itself, the difference between ordinary grout and expansive grout is mainly reflected in two aspects. The first is that during the development process, the ordinary grout will undergo dry shrinkage, resulting in the reduction of volume, while the volume of expansive grout will increase and produce greater stress due to the action of expansive agent. The second is the difference of mechanical strength. The porosity of expansive grout increases due to volume expansion. Therefore, under the same environment, the compressive strength of expansive grout is lower than that of ordinary grout. Expansive grout produces a corresponding expansion stress during the expansion process, but the increase in micro-cracks between crystals during the volume expansion process also leads to a decrease in the mechanical strength of the grout [19,20]. A CaO-based expansive agent will produce a large amount of Ca(OH)_2_ in the hydration reaction process, and the formation of Ca(OH)_2_ is the main reason for the expansion of expansive grout and is also the reason for a large number of microcracks and increased porosity in the stone body. The decrease in the mechanical strength of the expansive grout leads to a poor grouting effect, which in turn affects the overall strength of the rock mass. Therefore, the problem remains as to how the mechanical strength of the grout can be increased without affecting the expansion performance of the grout. To solve this problem, the inactive mineral admixture was used to fill the micro-cracks inside the grout. In engineering, quartz sand, an inactive mineral admixture [21,22,23], is often applied for enhancing the properties of grout [24,25]. Quartz sand is low cost and widely available, and possesses stable chemical properties, effectively not reacting with cement components. Therefore, it may be the most suitable strength-enhancing material for expansive grout [26,27].

Due to the lack of relevant research on expansive grout with quartz sand as the grouting material, the influence of quartz sand on the expansion and strength performance of expansive grout are still unknown. Therefore, we designed an orthogonal ratio test with different quartz sand and expansive agent contents, and the expansion ratios are monitored during the development of the samples. On this basis, uniaxial compression strength (UCS) tests are carried out to measure the mechanical strength of the samples under different proportions. Additionally, the influence laws of the mechanical strength on the quartz sand and the expansive agent are summarized. At the same time, acoustic emission monitoring is carried out during the uniaxial compression, because the failure processes of samples with different compositions may have different acoustic emission (AE) signal releasing behavior [28,29]. The development of micro-cracks in the various samples are analyzed according to the internal sound signals of the samples, and the mechanical strength of the different samples with different contents of expansive agent and quartz sand are studied. Finally, SEM and XRD tests are conducted to analyze the morphological characteristics and phase composition of the expansive grout, and the expansion and mechanical performance of samples with different contents of expansive agent and quartz sand on a microscopic level are also explained.

## 2. Experiment

### 2.1. Materials

The main materials in the tests were 42.5# ordinary Portland cement, 80–120 mesh quartz sand and high static crushing agent (HSCA). Their compositions are shown in Table 1.

The expansion mechanism of HSCA is mainly material transfer, solid phase volume expansion and pore volume growth; that is, CaO molecules react with water molecules to form Ca(OH)_2_ crystals, resulting in volume expansion and expansion pressure [13,14]. In addition, as the molecular volume increases, it is difficult for Ca(OH)_2_ to fully interweave with other reaction products. There are voids between the Ca(OH)_2_ crystals and other reaction products, which manifests as an increase in pore volume [30,31].

Quartz sand, as a mineral admixture, is introduced to improve the hydration degree of cement, increase the density of cement by filling the voids created by Ca(OH)_2_ crystals, and finally increase the strength of the grout. 

### 2.2. Proportion Test Schemes

In accordance with our previous study, a water–cement ratio of 0.7 was adopted, simultaneously taking into account the fluidity and expansion performance [13,14]. At the same time, considering the impact of quartz sand and the expansive agent on the expansion and mechanical performance of expansive grout, five levels (3%, 6%, 9%, 12% and 15%) of the expansive agent contents and four levels (0%, 5%, 10% and 15%) of the quartz sand were set. On this basis, the appropriate amount of flash setting admixture (FSA) and defoamer were added to increase cement cementation speed and reduce bubbles, respectively. The test was carried out at room temperature (25 °C) and the materials were mixed with tap water at 20 °C. The test scheme proportion is shown in Table 2.

### 2.3. Experiment Methods

**Figure 1:**
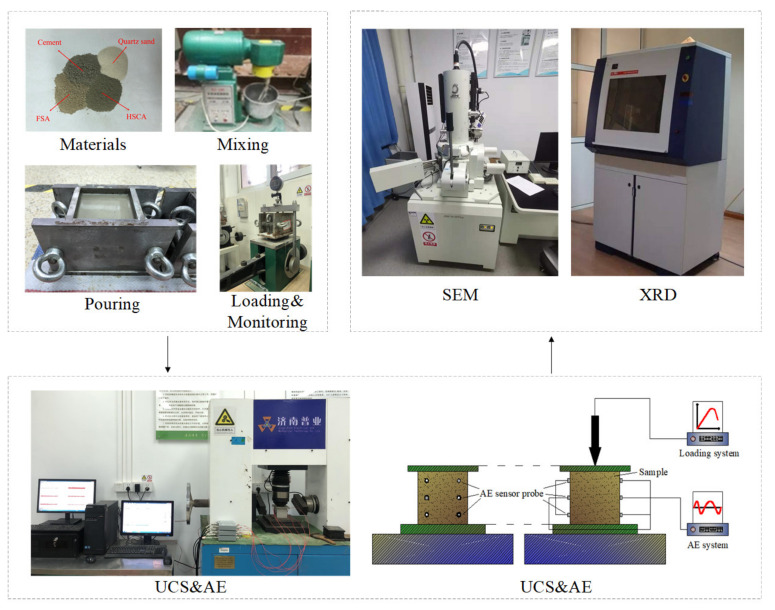
Test process.

**Figure 2:**
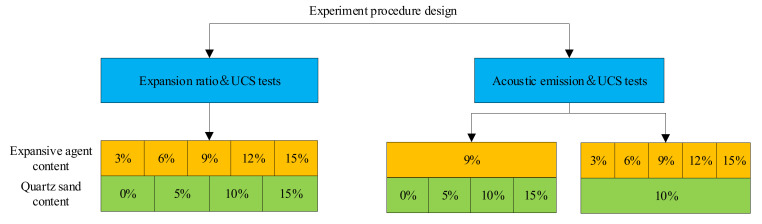
Test plan.

This test mainly includes volume expansion ratio test, UCS, AE, SEM and XRD. The specific test process is shown in Figure 1, and the test plan is presented in Figure 2.

#### 2.3.1. Sample Preparation

The raw materials were weighed according to the test scheme proportion. After adding the prepared raw materials into a mixer and mixing well for 10–20 minutes, the stirred grout was poured into the cubic steel mold with the size of 70.7 × 70.7 × 70.7mm. After the sample in the mold was vibrated on a vibrating table until all the bubbles were expelled, it was set in the consolidator when the sample reached the final setting, and an axial pressure of 0.5MPa was applied for 5 days.

#### 2.3.2. Volume Expansion Ratio

Due to the lateral constraints of the steel mold, the height change ratio of the sample is the volume expansion ratio of the sample. A dial gauge was used to measure the initial height $h_{0}$ of the sample after the final setting and the height h during the curing process, the volume expansion ratio of the sample was calculated using Formula (1).

(1) $$\phi =\frac{h-h_{0}}{h_{0}}\times 100\%$$

Because the volume expansion of the expansive grout mainly occurred rapidly in the early stages and hardly expanded further after 5 days, the height of the sample was monitored every 3 h on the first day, every 12 h on the second day and every 24 h thereafter.

#### 2.3.3. Uniaxial Compressive Strength

The UCS of the sample was tested by using a YZW–30A rock direct shear apparatus. The loading process was controlled by displacement and test force; the loading rate was 0.02 mm/s. The failure characteristics of the samples with different contents of quartz sand and expansive agent could be observed, and the stress–strain curves of the samples under uniaxial compression could be obtained.

#### 2.3.4. Acoustic Emission

An Express–8 acoustic emission monitoring system was used for monitoring the acoustic emission signal releasing. After polishing the layout area of the sample and smearing the couplant on the surface of the sensors, three sensors were set on both the left and right lateral side of the sample. The released acoustic emission signals were collected during the compression process.

#### 2.3.5. Scanning Electron Microscope and Microscope X-ray Diffraction

The sample with different contents of expansive agent and quartz sand were dried and ground into powders. The internal micro-cracks of the sample powders were observed using JSM–7610F field emission scanning electron microscope. 

A representative mix proportion group was selected for the XRD test. Group 1 was a sample with 10% quartz sand content and five levels of expansive agent content. Group 2 was a sample with 9% expansive agent content and four levels of quartz sand content. Qualitative phase analysis was determined by k-value method, which indicates the relative content of the phase.

In the SEM test, samples with 10% quartz sand content and five levels of expansive agent content were selected for the test, and the most representative mix proportion was selected to explain the filling mechanism of quartz sand.

## 3. Expansion Characteristics

### 3.1. Expansion Stage

**Figure 3:**
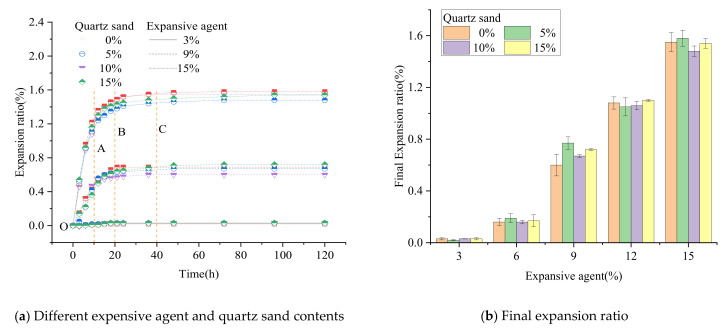
Expansion development of expansive grout samples.

The volume expansion development with different contents of expansive agent and quartz sand within 5 days is shown in Figure 3.

It can be seen from Figure 3a that the increasing trend of the sample volume is roughly the same, all of which increase rapidly and then slowly. According to the expansion trend, the expansion development process of the sample is divided into four stages:

Rapid expansion stage (O–A): The sample rapidly expanded to more than 70% of the final expansion ratio, which occurred within 10 h after final setting.

Slow expansion stage (A–B): After the rapid expansion stage, the expansion speed of the sample was significantly reduced, and it expanded at a slow speed to about 90% of the final expansion ratio. This stage occurred within 10 to 30 h after final setting.

Residual expansion stage (B–C): after the first two stages of expansion, the sample underwent residual expansion at a very low rate. The residual expansion value was between 1% and 10% of the final expansion ratio and occurred within 2 to 3 days after final setting.

Stable stage (C–): After the volume expansion developed for more than 3 days, it reached a basically stable state with no expansion.

### 3.2. Expansion Ratio

(1) Different expansive agent contents:

In the case of the same quartz sand content and load condition, the expansion development of the samples with different expansive agent contents are shown in Figure 3a. It can be seen from Figure 3b that the final expansion ratio of the sample is positively correlated with the expansive agent contents.

Taking the quartz sand content of 10% as an example, when the expansive agent contents were 3%, 6%, 9%, 12%, 15%, the final expansion ratio of the samples were 0.03%, 0.16%, 0.67%, 1.06%, 1.48%, respectively. The functional relationship between the final expansion ratio ρ and the expansive agent content ω% was calculated using Formula (2).

(2) ρ=0.127ω−0.460   R²=0.977

It can be seen from Equation (2) that the volume expansion ratio of the expansive grout is linear related to the expansive agent content.

(2) Different quartz sand contents:

In the case of the same expansive agent content and load condition, the expansion development of samples with different quartz sand content with time is shown in Figure 3a. It can be seen from Figure 3a,b that both the expansion development process and the final expansion ratio of samples with different quartz sand contents were quite similar, which means that the quartz sand had extremely little effect on the expansion behavior of expansive grout.

### 3.3. XRD

The macro expansion behavior and mechanical properties of grout with different expansive agent and quartz sand contents were revealed from XRD patterns and qualitative phase analysis. The main products of cement hydration are C–S–H gels and Ca(OH)_2_ crystals [32,33]. C–S–H is the main product which determines the strength of the grout. The higher the relative content of C–S–H, the greater the strength of the sample. Ca(OH)_2_ determines the volume expansion of the sample. The higher the content of Ca(OH)_2_, the greater the expansion ratio but also the greater the reduction in reduce the mechanical strength of the sample. The expansive grout has the same hydration products as ordinary cement, but its development process and phase composition are different from ordinary cement because of the effect of the expansive agent, and the porosity filling behavior of quartz sand in the expansive grout.

**Figure 4:**
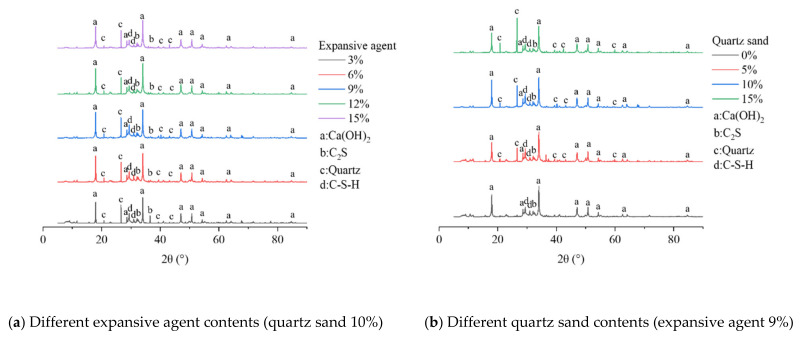
XRD patterns of expansive grout samples.

**Figure 5:**
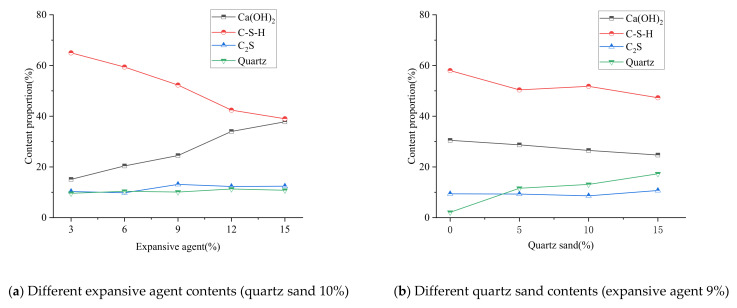
XRD phase compositions of expansive grout samples.

Figure 4 is the XRD patterns of the samples with different expansive agent and quartz sand contents, Figure 5 shows their qualitative phase analysis. We can observe in Figure 4 and Figure 5:

i. The diffraction patterns of the samples with different expansive agent and quartz sand contents were similar, and the hydration products were basically the same. The main hydration products were C–S–H, Ca(OH)_2_, and C_2_S, which were the same as ordinary cement hydration products. Quartz can be seen in the spectrum of the samples with quartz sand, but not in the samples without it.
ii. According to the peak value and peak area, C–S–H and Ca(OH)_2_ were the main hydration products of the grout, and their crystallinities and contents were both higher.
iii. With the same quartz sand content, as the expansive agent content increased, the relative contents of C–S–H and Ca(OH)_2_ showed an obvious downward and upward trend, respectively, but the contents of quartz sand and C_2_S remained relatively stable.
iv. With the same expansive agent content, as the content quartz sand increased, the contents of Ca(OH)_2_ and C–S–H decreased slightly, but the content of quartz shows an upward trend.

The main reasons for the difference of XRD phases caused by different expansive agent contents are because, as the expansive agent content increased, the CaO in the expansive agent participated in the hydration reaction and produced a large amount of hydration products; then, the content of Ca(OH)_2_ increased, which limited the generation of C–S–H [32,33].

The main reason for the quantitative difference in XRD phase caused by different quartz sand content is that the quartz sand led to a reduction in the relative quality of cement and expansive agent in the sample, resulting in the slight reductions of the relative contents of C–S–H and Ca(OH)_2_, but their development was not limited by quartz. The chemical properties of quartz sand were stable, and it did not effectively participate in the hydration reaction; therefore, the quartz content increased along with the quartz sand content increase. The expansion ratio of the expansive grout was mainly determined by the content of Ca(OH)_2_; the macro volume expansion of the sample was hardly affected by quartz sand because there were extremely few changes in its content. 

## 4. Mechanical Behavior

### 4.1. UCS

**Figure 6:**
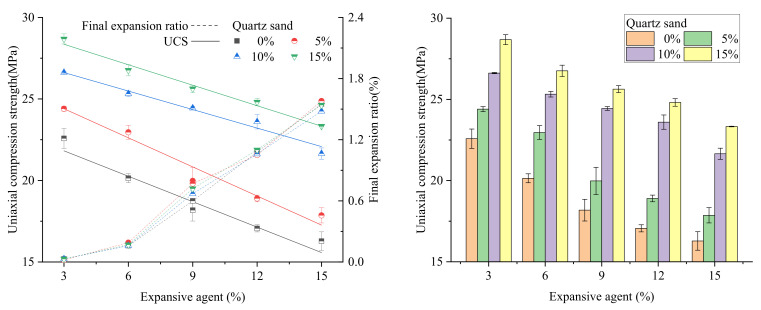
The UCS of expansive grout samples.

The UCS of the samples with different expansive agent and quartz sand contents are shown in Figure 6.

It can be seen from Figure 6 that with the same content of quartz sand, the UCS of the samples decreases with the increase in the expansive agent content. In the case of the same expansive agent content, the UCS of the sample increases with an increase in the quartz sand content.

Under the condition of different expansive agent contents, compared with the UCS of the sample with no quartz sand, when the quartz sand contents were 5%, 10%, 15%, the UCS increased by 10.51%, 29.88%, and 37.92% on average, respectively. The strength enhancement effect of the expansive grout can be clearly observed.

The functional relationship between strength σ and expansive agent contents ω% and quartz sand contents  ε% can be calculated using Formula (3).

(3) σ=23.070−0.476ω+0.450ε 

### 4.2. Stress–Strain Curves

Because the failure characteristics are similar, two representative groups of experiments were selected to analyze the failure characteristics. In Group 1, the content of quartz sand is 10%, and the content of expansive agent is the variable. In Group 2, the content of expansive agent is 9%, and the content of quartz sand is the variable.

**Figure 7:**
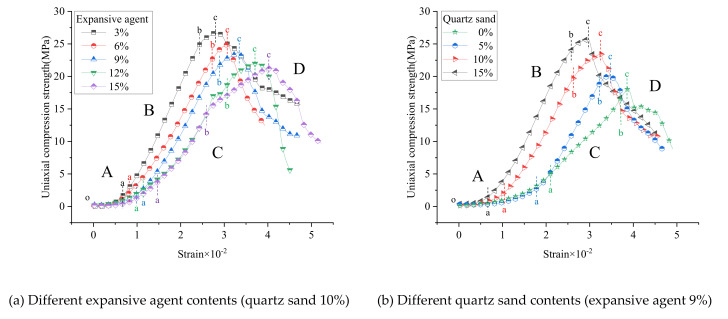
Stress–strain curves of expansive grout samples.

The uniaxial compression stress–strain curves of the expansive grout samples with different expansive agent and quartz sand contents are shown in Figure 7.

It can be seen from Figure 7 that the uniaxial compression stress–strain curve of the samples could be divided into four phases: A (o–a): the initial pore compaction stage, B (a–b): the linear elastic stage, C (b–c): the yield stage, and D (c–): the post-peak failure stage [13,14]. The expansive grout is a typical elastoplastic material; however, with different expansive agent and quartz sand contents, the stress–strain performance in some stages have significant differences.

(1) Different expansive agent contents:

In the case of the same quartz sand content (10%), the uniaxial compression stress–strain curves of the expansive grout samples with different expansive agent contents are shown in Figure 7a.

i. The deformation generated during the uniaxial compression failure of the sample increased with the increase in the expansive agent content. For the lesser content of expansive agent, the deformation was lower before the sample reached its peak strength.
ii. The elastic modulus of the expansive grout sample decreased with the increase in the expansive agent content. In the linear elastic stage, for the lesser content of expansive agent, the tangent slope of the straight-line segment of the curve was greater, i.e., the tangent modulus of the sample was higher.
iii. The deformation of the expansive grout sample in the yield and post-peak stages increased with the increase in the expansive agent content.

The specific reason for the characteristics of the above uniaxial compression stress–strain curve is that for a higher content of expansive agent, more micro-cracks were produced during the sample volume expansion. In the process of uniaxial compression, the macroscopic analysis showed that more deformation developed in the initial pore compaction stage, where the elasticity was weaker, but the plasticity was stronger; therefore, the elastic modulus was lower. The stiffness and brittleness of the sample were weaker, which made the sample easier to deform and caused its strength to be lower.

(2) Different quartz sand contents:

In the case of the same expansive agent content (9%), the uniaxial compression stress–strain curves of the expansive grout samples with different quartz sand contents are shown in Figure 7b.

i. The deformation of the expansive grout sample in the initial pore compaction stage decreased with the increase in the quartz sand content. For higher quartz sand content, the deformation of the sample was lower in this stage.
ii. The deformation of the expansive grout sample before the peak point decreased with the increase in the quartz sand content. For the higher quartz sand content, the deformation was lower before the sample reached the peak strength.
iii. The elastic modulus of the expansive grout sample increased with the increase in the quartz sand content. In the linear elastic stage, for higher expansive agent content, the tangent slope of the straight-line segment of the curve was greater, i.e., the tangent modulus of the sample was higher.
iv. The deformation of the expansive grout sample in the yield and post-peak stages decreased with the increase in the quartz sand content.

The specific reason for the characteristics of the above uniaxial compression stress–strain curve is that for higher quartz sand content, the filling effect of quartz sand on the micro-cracks of the sample caused by volume expansion was more obvious. In the process of specimen uniaxial compression, macroscopically, the deformation in the initial pore compaction stage was lower and the elastic modulus of the specimen was higher. The stiffness and brittleness of the sample were stronger, which meant the sample was harder to deform and that the strength was greater.

### 4.3. Failure Characteristics

**Figure 8:**
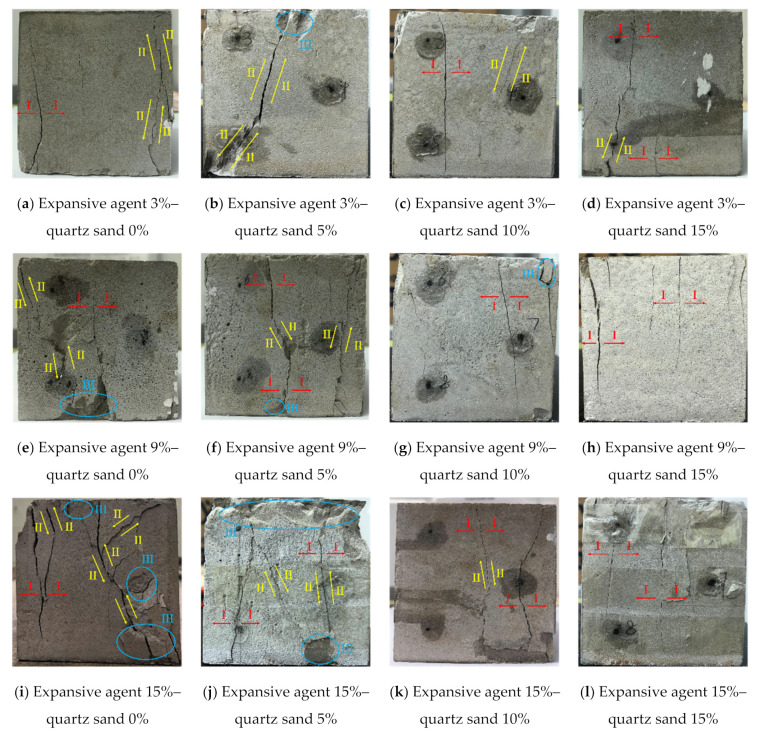
Failure modes of expansive grout samples under uniaxial compression.

The uniaxial compression failure characteristics of the samples with different expansive agent and quartz sand contents are shown in Figure 8. According to the rock mechanics theory, the failure modes of rock are generally divided into tensile failure, shear failure and tensile–shear composite failure [34,35], and the failure cracks are mainly tensile cracks (Type I), shear cracks (Type II) and tensile–shear cracks. During the failure process, the sample may also have flake crack, which is defined as Type III. According to the different failure characteristics, the mechanical properties of expansive grout samples with different expansive agent and quartz sand contents could also be revealed.

Figure 8 shows that in the case of the same expansive agent content, with the gradual increase in quartz sand content, the transformation from Type II failure to Type I failure was more obvious, and Type III cracks gradually decreased. The number of cracks basically decreased, especially when the expansive agent content was high.

The main reasons for the above characteristics are as follows: with the same content of expansive agent, for higher quartz sand content, the filling effect of the internal pores of the samples was better, resulting in a higher internal density. The expansive grout sample transformed from ductility to brittleness, and its failure mode transformed towards tensile failure (Type I). Furthermore, when the content of the expansive agent was high, there were more micro-cracks inside the sample was filled with quartz sand, so the filling effect was more obvious.

### 4.4. SEM

**Figure 9:**
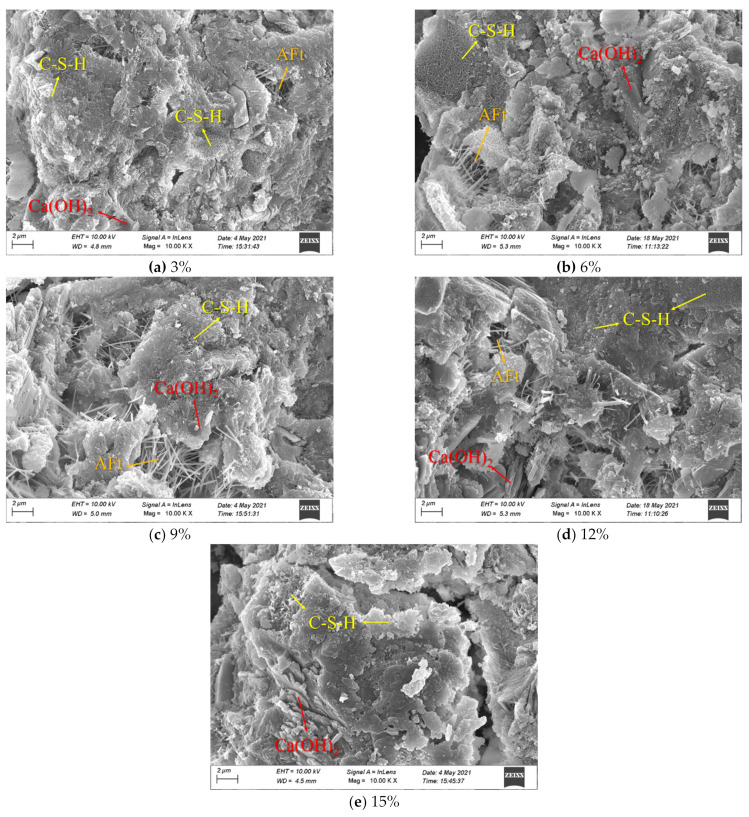
SEM micrographs of samples with different expansion agent contents (quartz sand 10%).

**Figure 10:**
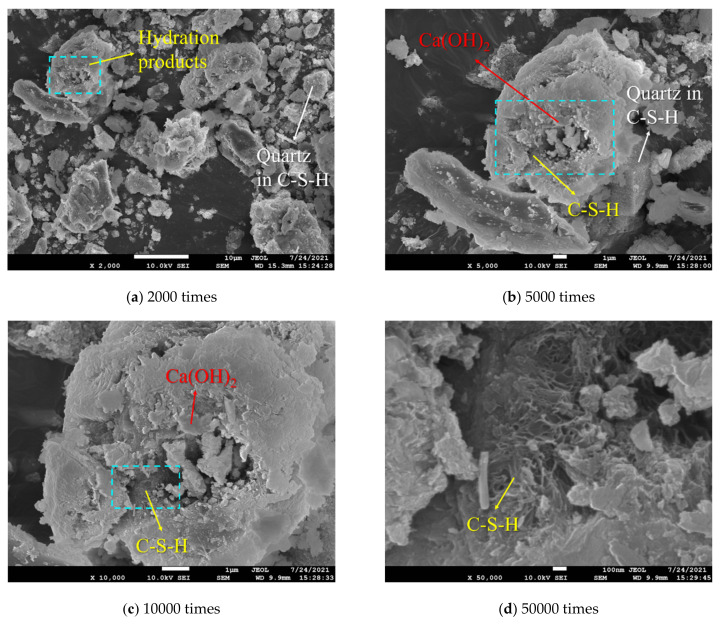
SEM micrographs of the sample having 9% expansive agent and 10% quartz sand at different magnifications.

Figure 9 shows the SEM micrographs of the samples with different expansive agent contents, while Figure 10 shows the SEM micrographs of the samples with different magnification when their quartz sand contents are 10%.

It can be observed in Figure 9 that the main hydration products of expansive grout were flocculent–type C–S–H gels, hexahedron–type Ca(OH)_2_ crystals, needle–type Aft(ettringite) crystals [32,33]. With the increase in expansive agent content, CaO in the expansive agent participated in the hydration reaction and produced a large number of hydration products, while the number of Ca(OH)_2_ crystals became larger and formed extrusion to C–S–H gels with certain strength, resulting in more pores in the internal structure and a reduction in compactness. Macroscopically, this manifested as volume expansion and a reduction in strength in the expansive grout [14,17].

It can be seen from Figure 10 that when the samples contained quartz sand, the main hydration products were still C–S–H and Ca(OH)_2_, which revealed similar characteristics to the XRD patterns of the expansive grout samples (see Figure 4). The surface of quartz was covered by a large amount of C–S–H gels and Ca(OH)_2_ crystals and had a good filling effect on the internal pores of the sample. 

**Figure 11:**
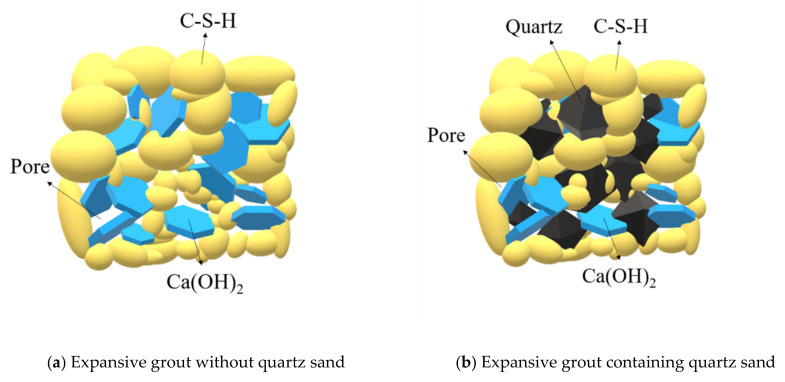
Strength enhancement mechanism of quartz in expansive grout.

Figure 10 shows the macro expansion behavior and strength properties of the expansive grout from the micro level. Quartz does not react in the hydration process of expansive grout or limit the development of Ca(OH)_2_ crystals; however, it does fill the internal micro pores and cracks. Therefore, the expansion ratio of the sample does not change significantly, but the compactness of the sample increases, resulting in an increase in the samples’ UCS. According to the above analysis, the strength enhancement mechanism of quartz sand in the expansive grout is shown in Figure 11.

## 5. AE Characteristics

**Figure 12:**
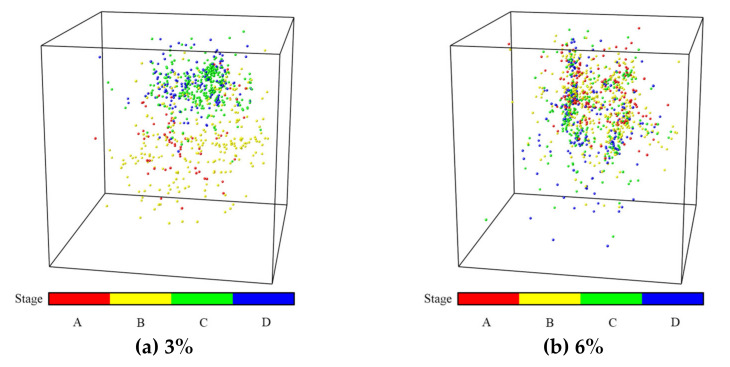

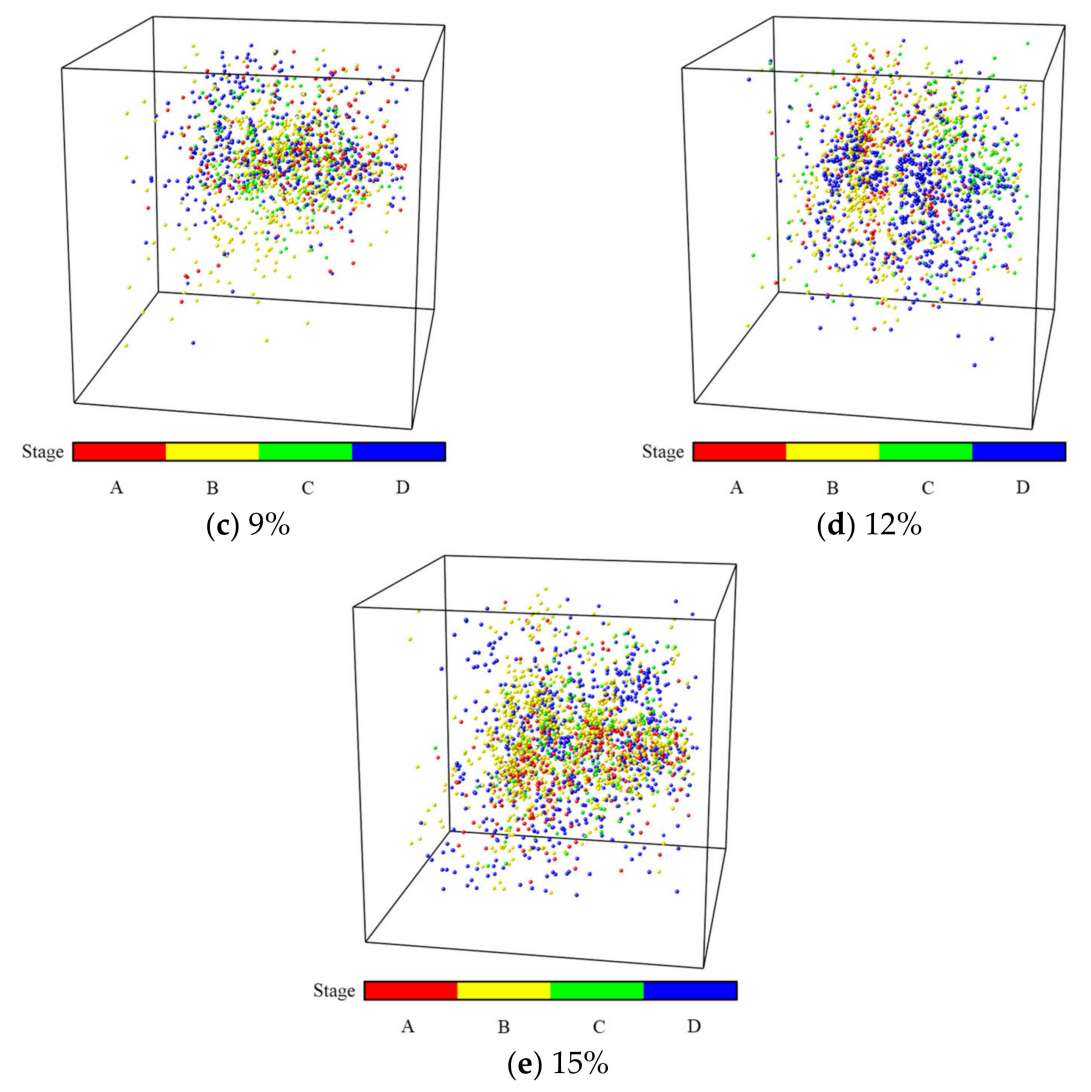
Acoustic emission damage location diagram of samples with different expansive agent content (quartz sand 10%) in different failure stages (A–D).).

**Figure 13:**
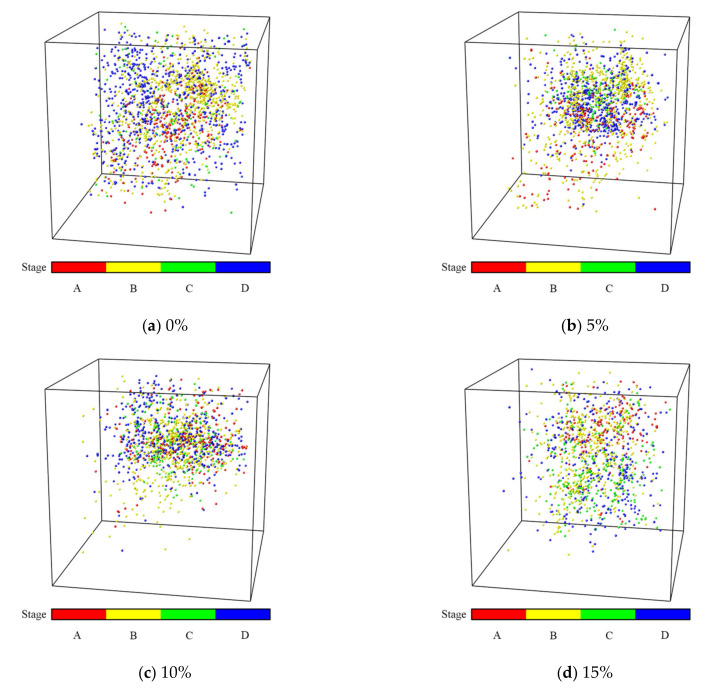
Acoustic emission damage location maps of samples with different quartz sand contents (expansive agent 9%) in different failure stages (A–D).)

**Figure 14:**
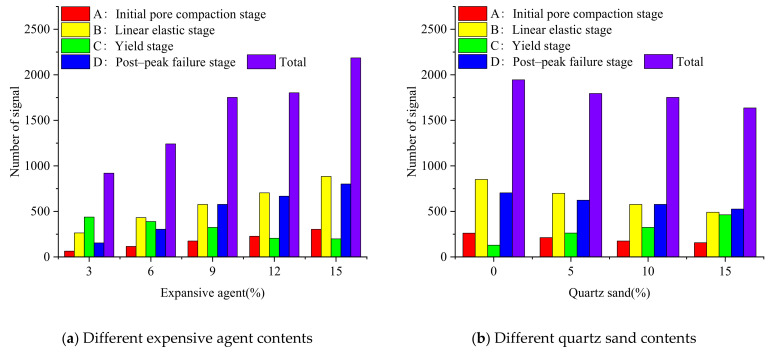
The number of acoustic emission signals in different failure stages (A–D).).

**Figure 15:**
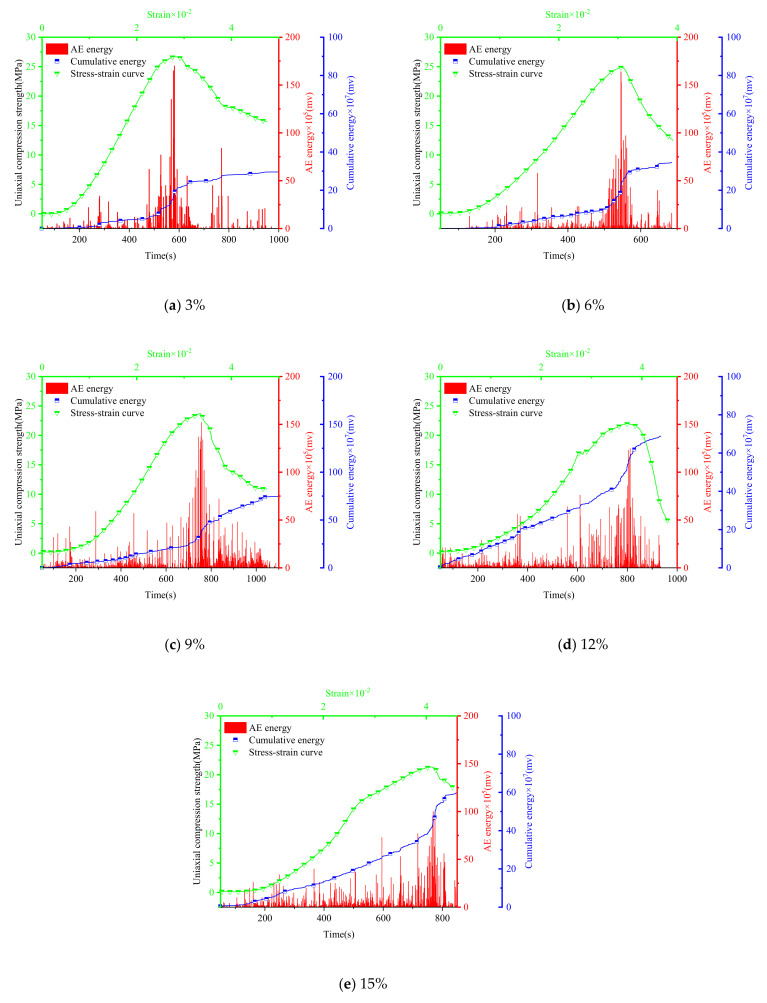
Acoustic emission energy diagrams of samples with different expansive agent contents (quartz sand 10%).

**Figure 16:**
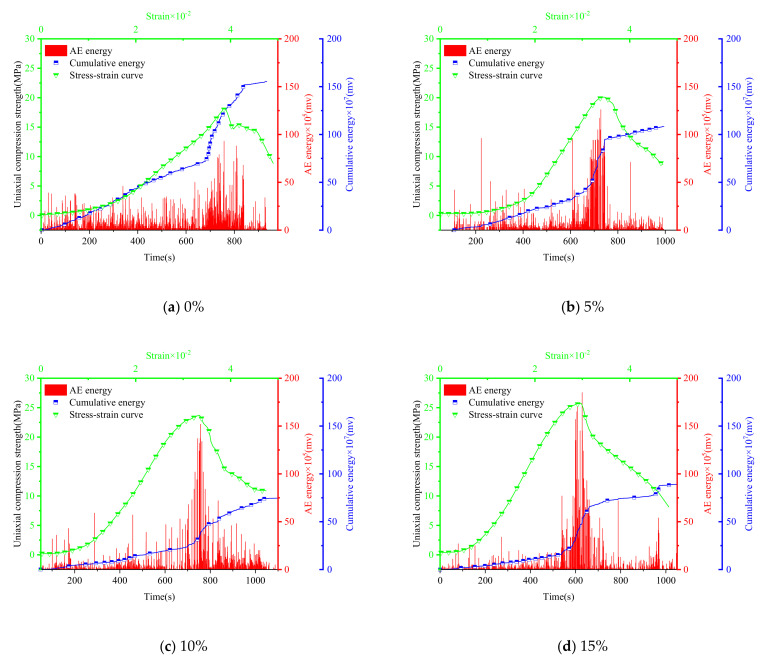
Acoustic emission energy diagrams of samples with different quartz sand contents (expansive agent 9%).

The AE damage location signal points (Figure 12 and Figure 13), the numbers of acoustic signals in different stages (Figure 14) and the energy of AE signal points (Figure 15 and Figure 16) were obtained from uniaxial compression AE test of the sample with different expansive agent and quartz sand contents. According to the division of uniaxial compression stress–strain curves (in Section 4.2), the AE points in four stages were marked with different colors correspondingly. In Figure 12 and Figure 13, the initial pore compaction stage, the linear elastic stage, the yield stage, and the post-peak failure stage was represented by stage A, B, C, and D, respectively. The four stages correspond to the failure characteristics of Figure 7. 

It can be seen from Figure 12 and Figure 13 that with different expansive agents and quartz sand contents, the locations of the samples did not change significantly, and the spatial locations of the events were obviously discrete, indicating the micro damages were evenly distributed throughout the sample. In addition, AE events nucleate and form clusters, and the damage points were more concentrated in the rough part of the cracks [36,37].

Although there was no obvious change in the distribution of AE points inside the sample, it can be seen from Figure 14 that the number of AE points in different stages were significantly different. In the initial compaction stage (A) and linear elastic stage (B), these two stages are mainly the shrinkage of initial micro-cracks and the generation of fresh cracks in the rock sample. The more acoustic emission signal points, the more internal initial cracks, and the better the plasticity of the sample. In the yield stage (C), the failure of the rock samples, the large number of plastic strains increase sharply, and the greater acoustic emission signal points all show that the more elastic waves generated during failure, the more concentrated signal points in this stage, and the worse the plasticity of the sample. Furthermore, the AE energy of the samples with different expansion agent and quartz sand contents during the uniaxial compression process were also different (see Figure 15 and Figure 16). 

According to Figure 14, Figure 15 and Figure 16, the acoustic emission law of samples with different expansion agent and quartz sand contents can be summarized as follows.

i. The total number of AE signals and the number of them in initial pore compaction stage, linear elastic stage and post-peak failure stage increased with the increase in the expansion agent content, but the number of them in yield stages decreased. However, with the increase in the quartz sand content, the change laws of the total number of AE signals and the number of them in the four different stages were just the opposite of those with the increase in the expansion agent content.
ii. With the increase in the expansion agent content, the AE energy distribution of the sample was more dispersed, mainly in the linear elastic and the post-peak failure stages. At the same time, the energy amplitude was lower during the yield stage, but the variance of energy at different times was lower during the uniaxial compression process. The accumulative energy increased overall.
ii. The influence of quartz sand on AE energy was also opposite to that of the expansion agent. With the increase in the quartz sand content, the AE energy distribution of the sample was more concentrated, the energy in the linear elastic stage and the post-peak failure stage were reduced. More energy was concentrated in the yield stage, and the energy amplitude was higher. The variance of the energy at different times was higher, and the accumulative energy generally showed a downward trend.

The main explain for AE characteristics due to different expansion agent content could be summarized as follows. For higher expansion agent content, more micro-cracks were generated, which caused the internal porosity to increase during the volume expansion, resulting in a decrease in internal compactness. In the initial pore compaction and linear elastic stages, a large number of initial micro-cracks were closed and slipped, so the signal points increased during these periods. The amplitude of the energy during the yield stage of the sample decreased, which indicated that the stiffness of the sample decreased as well as the instantaneous energy value of the failure point. In addition, the increase in the number of signals and the energy in the post-peak failure stage also showed that the sample transferred from brittleness to ductility in the macroscopic view, and the residual stress acted longer after the sample had totally yielded. Therefore, with the increase in the expansion agent content, although the energy in the yield stage was reduced, the energy in the other three stages was greater, and more energy was generated in the pore compaction phases and the ductile failure phase after the peak strength of the sample; therefore, the overall accumulative energy was higher.

On the contrary, due to the filling effect of quartz sand on the internal pores of the expansion grout sample, for higher quartz sand content, fewer initial micro-cracks were generated in the sample and the compactness of the sample was higher. Therefore, the influence of the quartz sand on the AE characteristics was the opposite of those of the expansion agent. The reason for this was also no doubt the opposite, based on the above analysis.

## 6. Conclusions

In order to effectively improve the strength of the expansive grout, ultrafine quartz sand is used as the added aggregate. The volume expansion ratio, UCS, AE, SEM and XRD tests of samples were carried out to analyze the expansion behavior and strength properties of the expansive grout with quartz sand. According to the experimental results, the following main conclusions are drawn:

1. The expansion ratio and stages of the expansive grout are not affected by quartz sand. The reason is that the main component of quartz sand is silica, which has stable chemical properties and does not effectively participate in the hydration reaction.
2. Adding quartz sand to the expansive grout can effectively increase its strength. For higher quartz sand content, the UCS of the sample is greater. Compared with not adding quartz sand, when the content of quartz sand is 5%, 10%, and 15%, the UCS increases by 10.51%, 29.88%, and 37.92%, respectively. Additionally, for higher content of expansive agent, the enhancing effect of quartz sand is more significant.
3. Quartz sand does not affect the growth of Ca(OH)_2_ crystals that determine the expansion of the grout, so it does not affect the expansion behavior of the expansive grout macroscopically. Furthermore, the micro-cracks caused by the expansion process of expansive grout are filled by the quartz, and the compactness of the grout is improved, so the UCS of the sample increases in the macroscopic view. It can be seen that quartz sand is a good strength-enhancing additive material for expansive grout.

## Figures and Tables

**Table 1:** Main chemical compositions of grout materials. wt%.

Material	CaO	SiO_2_	SO_3_	Fe_2_O_3_	Al_2_O_3_	MgO	K_2_O	TiO_2_	Na_2_O
Portland cement	44.00	30.12	2.05	2.05	11.66	4.74	1.07	0.29	1.73
HSCA	87.12	4.47	0.04	2.76	2.78	0.75	0.06	0.09	0.21
Quartz sand	–	99.30	–	0.02	0.3	–	–	–	–

**Table 2:** Test scheme proportion.

Water–Cement Ratio	Expansive Agent (%)	Quartz Sand (%)	Flash Setting Admixture (%)	Defoamer (%)
0.7:1	3/6/9/12/15	0/5/10/15	2.5	0.2

Note: All percentages in the table except for quartz sand are based on the sum of the mass of cement and water, and the content of quartz sand is based on the mass of cement.

## Data Availability

No data, models, or code were generated or used during the study (e.g., opinion or data less paper).
